# Examining changes in the prevalence of cost‐motivated alcohol reduction attempts in the context of a cost‐of‐living crisis and alcohol duty reforms: A population survey of risky drinkers in Great Britain, 2021–2024

**DOI:** 10.1111/add.70248

**Published:** 2025-11-19

**Authors:** Sarah E. Jackson, Jamie Brown, Colin Angus, Abi Stevely, Magdalena Opazo Breton, Leonie Brose, Luke Wilson, John Holmes

**Affiliations:** ^1^ Department of Behavioural Science and Health University College London London UK; ^2^ SPECTRUM Consortium Edinburgh UK; ^3^ Sheffield Centre for Health and Related Research (SCHARR) University of Sheffield Sheffield UK; ^4^ School of Medicine University of Nottingham Nottingham UK; ^5^ Addictions Department Institute of Psychiatry, Psychology and Neuroscience, King's College London London UK

**Keywords:** alcohol consumption, alcohol duty, alcohol reduction attempts, alcohol tax, cost‐of‐living crisis, increasing and higher risk drinking

## Abstract

**Background and aims:**

Affordability of alcohol is a key driver of consumption. The cost‐of‐living crisis in Great Britain has been putting pressure on household budgets since late 2021. In addition, the UK Government implemented substantial reforms to the alcohol duty system and increased alcohol taxes in 2023. This study aimed to estimate changes in the monthly prevalence of cost‐motivated alcohol reduction attempts among risky drinkers over this period.

**Design:**

Data were drawn from the Alcohol Toolkit Study, a nationally representative monthly cross‐sectional household survey.

**Setting:**

Great Britain.

**Participants:**

26 212 risky drinkers [alcohol use disorders identification test – consumption (AUDIT‐C) score ≥5] aged ≥18y surveyed between January 2021 and December 2024 [mean (SD) age = 45.9 (17.1); 61.4% men].

**Measurements:**

The primary outcome was having tried to reduce alcohol consumption in the past year due to a decision that drinking was too expensive (‘cost‐motivated alcohol reduction attempt’). This included participants who also reported other motives (e.g. health concerns) for trying to reduce their consumption.

**Findings:**

Overall, 1355 participants reported making a cost‐motivated alcohol reduction attempt. The monthly weighted prevalence of cost‐motivated alcohol reduction attempts among risky drinkers increased from 4.6% in January 2021 to 7.0% in December 2024 [prevalence ratio (PR) = 1.54, 95% confidence interval (CI) = 1.34–1.74]; equating to ~1.1 million people attempting to reduce their drinking among risky drinkers in 2024. This was primarily driven by a rise in the proportion of all alcohol reduction attempts that were motivated by cost, from 12.4% to 19.7% (PR = 1.58, 95% CI = 1.39–1.77), rather than an overall increase in the prevalence of alcohol reduction attempts (which remained relatively stable across the period at an average of 36.0%). The pattern of results was similar when the outcome was restricted to alcohol reduction attempts *only* motivated by cost [17.3% (95% CI = 15.0–19.7%) of all cost‐motivated alcohol reduction attempts].

**Conclusions:**

During a period of increasing financial pressures in Great Britain, alcohol reduction attempts were increasingly motivated by cost but the overall prevalence of reduction attempts did not increase.

## INTRODUCTION

Alcohol use is a leading preventable risk factor for disease and premature death [1, 2], with greater risks for those who drink more heavily [3]. There have been large increases in alcohol‐specific deaths in the UK in recent years, rising by 38.4% between 2019 and 2023 [4], with the highest rates observed among more deprived groups [5]. This appears to have been driven by a sharp increase in the proportion of adults drinking at risky levels since the COVID‐19 pandemic [5], defined as a score of 5 or higher (out of a possible 12) on the Alcohol Use Disorders Identification Test – Consumption (AUDIT‐C) scale (henceforth referred to as ‘risky drinking’) [6]. At the start of the pandemic, the prevalence of risky drinking among adults in England increased from around one in four to around one in three, and has remained relatively stable up to mid‐2025 [7]. There is therefore an urgent need to reduce alcohol consumption to improve public health and reduce inequalities.

Increasing the price of alcohol to reduce affordability is widely considered to be one of the most effective strategies for encouraging people to reduce their drinking and for reducing alcohol‐related harm [8, 9, 10, 11]. This is usually achieved through taxation. Considerable evidence shows that when alcohol prices or taxes increase, levels of alcohol consumption, risky drinking and heavy episodic drinking fall [10, 12, 13, 14, 15]. This type of price responsiveness is seen across all types of alcoholic beverages and levels of drinking [10]. There is also evidence that increasing prices leads to reductions in alcohol‐related deaths and other harms [11]. Besides tax increases, the affordability of alcohol may also reduce when economic pressures force people to reconsider their spending [16]. Alcohol may be something that people cut back on to reduce household outgoings [17]. Conversely, financial difficulties are a trigger for psychological distress [18], which can exacerbate existing alcohol problems and excess consumption [17].

Over the past few years, there have been two key factors that may have caused more people in Great Britain to try to reduce their alcohol consumption for financial reasons. First, since late 2021 a ‘cost‐of‐living’ crisis has been underway, marked by sustained increases in the prices of essential goods and services (such as food, energy and housing), driven by high inflation and rising interest rates, which have outpaced wage growth and eroded real‐terms disposable incomes [19]. This crisis has put pressure on household budgets, particularly among certain population subgroups (e.g. those on lower incomes, unemployed or with dependent children) [20, 21]. Although inflation has begun to decline more recently, many households continue to face elevated living costs, and there is no clear consensus on when the crisis may be considered to have ended. During the crisis, the prices of alcoholic products have risen more slowly than other food and drink categories [22], but the combination of more expensive alcohol and wider financial pressures may have prompted more people to cut down on their drinking. Second, substantial reforms to the alcohol duty system were implemented by the UK Government in August 2023 [23, 24]. The new system was designed to simplify and rationalise the duty system, in part to support public health. The most significant change was to tax all drinks, rather than just beer and spirits, in proportion to their alcoholic strength (alcohol by volume) as a disincentive to producing and purchasing stronger drinks. However, this principle was not applied consistently: for example, although cider is now taxed by strength, it is still taxed at a lower rate than equivalent‐strength beer. Furthermore, the introduction of the strength‐based taxation of wine was delayed until February 2025. Although strength‐based taxation is an important principle, the effects of the reforms on alcohol prices and therefore consumption are estimated to be minor [25]. In large part this is because the duty reforms themselves were designed to be close to revenue neutral for the government, with increased duty on wines largely offset by cuts to duty on pre‐mixed drinks and on beer and cider sold in pubs through the ‘draught relief’ mechanism [26]. However, subsequent evidence has suggested that the alcohol industry has responded by reducing the strength of some products, particularly beer, in response to incentives introduced in the new system for beers at lower strength (<3.5% alcohol by volume, ABV) [27]. More consequentially, in August 2023 the government also raised alcohol taxes by 10.1%, in line with inflation [24], while the cost‐of‐living crisis reduced household real‐terms disposable incomes.

We previously explored the impact of the first year of the cost‐of‐living crisis on the proportion of alcohol reduction attempts made by risky drinkers that were motivated by cost [28]. Overall, we observed an uncertain increase, from 12.0% of reduction attempts in December 2021 to 16.3% in December 2022. Analyses by socio‐economic position revealed that this was driven by changes among drinkers who were less advantaged, among whom this proportion doubled (from 15.3% to 29.7%), with little change reported among the more advantaged group. It is unclear how trends have continued to evolve since 2022, particularly in the context of the duty reforms, which may have provided additional financial incentives to cut down. It is also unclear whether trends have differed across other population subgroups or across nations. Within Great Britain, Scotland and Wales have a minimum unit pricing policy (i.e. a minimum price per unit of alcohol sold, where 1 UK unit = 8 g/10 ml), which prevents the sale of very low‐price alcohol and may therefore further influence the prevalence of cost‐motivated alcohol reduction attempts, whereas England does not. Scotland increased their minimum unit price from 50p to 65p in September 2024.

Understanding how motives for trying to reduce alcohol consumption are changing over time, and within which groups, can inform the development of targeted interventions to support behaviour change and reduce alcohol‐related harm. Rather than seeking to isolate the effects of the cost‐of‐living crisis, duty reforms and tax increases, we conceptualise these as interacting components of a complex and evolving policy and economic context that collectively shapes alcohol consumption. This approach reflects the multifaceted nature of both fiscal policy and economic pressures, the variability in implementation across nations, and the diverse ways in which industry and consumers may respond to contextual changes.

The Alcohol Toolkit Study has been collecting data on alcohol reduction attempts from a representative sample of adults in Great Britain regularly since before the cost‐of‐living crisis started. It is therefore well placed to provide up‐to‐date descriptive information on the levels of cost‐motivated reduction attempts and insight into trends over the entirety of this unstable period, to date. This study used these data to estimate time trends in the prevalence of cost‐motivated alcohol reduction attempts among risky drinkers and to explore differences by key potential moderators. Specifically, we aimed to address the following research questions:

1. How has the prevalence of cost‐motivated alcohol reduction attempts changed since January 2021 among (a) risky drinkers and (b) risky drinkers who made one or more attempts to reduce their alcohol consumption in the past year?
2. To what extent have any changes differed by level of risky drinking, nation, age, gender, socio‐economic position (indexed by occupational social grade), working status, children in the household, smoking status and psychological distress?

## METHODS

### Pre‐registration

The study protocol, research questions and analysis plan were pre‐registered on the Open Science Framework (https://osf.io/cp5d7/). After running the analyses, we simplified the categorisation of nation, social grade, working status, children in the household and psychological distress for the logistic regression models to provide more easily interpretable trends (because of the reduced number of subgroups). Trend results using the pre‐registered categorisations (see protocol) are provided in Appendix S1.

### Design

Data were drawn from the Alcohol Toolkit Study, an ongoing monthly cross‐sectional survey of a representative sample of adults (aged ≥16 years) in Great Britain [29, 30]. The study was established in 2014, and since 2020 uses a hybrid of random probability and simple quota sampling to select a new sample of approximately 2450 adults each month. The telephone interviews conducted by Ipsos MORI (London, UK) are conducted by landline and mobile phone using standard landline random digit dialling (RDD), mobile RDD and targeted mobile. Each eligible landline telephone number across Great Britain has a random probability of selection proportionate to population distribution and the mobile sampling is in proportion to the known mobile network share. Mobile, targeted mobile and landline sampling are carried out in approximately equal proportions. To maximise the response rates more landline sampling takes places earlier in the day, with more mobile sampling performed later in the day. Targeted mobile sampling relies on Ipsos MORI data about the likely characteristics of potential participants, based on age, location, sex, income and other demographic characteristics. These participants are targeted to fulfil quotas on the likelihood of answering. Therefore, unlike random probability sampling, it is not appropriate to record the response rate. While in theory it is possible for a participant to be included in more than one wave, this is very unlikely given the numbers sampled (we cannot determine whether any such cases exist because all data are fully anonymised). Comparisons with other national surveys indicate the survey achieves a nationally representative sample [31].

The present analyses focused on data from respondents surveyed between January 2021 and December 2024 (the most recent data at the time of analysis) who reported drinking at risky levels (defined as a score of ≥5 on the three‐item AUDIT‐C [32]). We selected January 2021 as the starting point to establish a baseline period prior to the onset of the cost‐of‐living crisis in late 2021.

Data were not collected from 16‐ and 17‐year‐olds in 2021, so we restricted the sample to age ≥18 years (the legal age of sale for alcohol in Great Britain) for consistency across the time series. In addition, since April 2022, alcohol reduction attempts have not been assessed in England in each monthly wave, so we include data only from the 36 waves that captured this variable (January–December 2021; January–April, June, August and October 2022; January–June, August, October and December 2023; January–April, June, August, October and December 2024).

### Ethics approval

Ethical approval for the Alcohol Toolkit Study was granted originally by the University College London (UCL) Ethics Committee (ID 0498/001). Participants provide informed consent to take part in the study, and all methods are carried out in accordance with relevant regulations. The data are not collected by UCL and are anonymised when received by UCL.

### Measures

#### Outcome

Alcohol reduction attempts were assessed with two questions: Q1, ‘How many attempts to restrict your alcohol consumption have you made in the last 12 months (e.g. by drinking less, choosing lower strength alcohol or using smaller glasses)? Please include all attempts you have made in the last 12 months, whether or not they were successful, and any attempt that you are currently making’; and Q2, ‘Are you currently trying to restrict your alcohol consumption, e.g. by drinking less, choosing lower strength alcohol or using smaller glasses?’ Those who responded ≥1 to Q1 or ‘yes’ to Q2 were considered to have made at least one past‐year alcohol reduction attempt. Although Q1 refers to the past 12 months, responses were collected monthly, allowing us to capture rolling trends in past‐year behaviour throughout the period from January 2021 to December 2024.

Those who reported making at least one past‐year alcohol reduction attempt were then asked: ‘Which of the following, if any, do you think contributed to you making the most recent attempt to restrict your alcohol consumption?’ Participants could select multiple motives from a list of options. Those who responded ‘A decision that drinking was too expensive’ were considered to have made a cost‐motivated alcohol reduction attempt.

#### Time

Survey month was analysed as a continuous variable, coded from January 2021 = 1 through December 2024 = 48, and modelled non‐linearly (see analyses). This coding included months with no data collection; models effectively interpolated estimates for these months at the aggregate level using information before and after the missing time points.

#### Potential moderators

Level of risky drinking (operationalised as the participant's AUDIT‐C score, with a possible range of 5–12) and age were analysed as continuous variables and modelled non‐linearly (see analyses). Nation was categorised as England versus Wales or Scotland. Gender was self‐reported as man versus woman; those who identified in another way were included in the analytic sample but were excluded from the analyses by gender owing to low numbers. Occupational social grade was categorised based on National Readership Survey classifications [33] as ABC1 (includes managerial, professional and upper supervisory occupations) versus C2DE (includes manual routine, semi‐routine, lower supervisory, state pension and long‐term unemployed). Working status was categorised as full‐time employment or self‐employed versus part‐time employment, unemployed and seeking work, or other. Children in the household was categorised as 0 versus ≥1. Smoking status was categorised as current, former or never smoking. Psychological distress was assessed using the Kessler Psychological Distress Scale (K6), which measures non‐specific psychological distress in the past month (with a possible range of 0–24) [34, 35]; we coded scores of ≤4 as no or low distress versus 5–12 as moderate distress and ≥13 as severe distress [34, 36]. Psychological distress questions were asked to all participants in England and to approximately 50% of participants in Wales and Scotland (owing to the availability of funding) up to June 2023; analyses using this variable were therefore limited to this period.

### Statistical analysis

Data were analysed in R 4.2.2 [37]. Missing cases (including non‐response and cases where variables were not assessed, i.e. the approximately 50% of those surveyed in Wales and Scotland not asked questions on psychological distress) were excluded on a per‐analysis basis. Levels of missing data were low across key variables and the prevalence of past‐year alcohol reduction attempts (overall and those motivated by cost) was similar between those with and without missing data (Appendix S2). Complete case analysis was deemed appropriate given the descriptive focus of the study and the minimal risk of bias.

The Alcohol Toolkit Study uses raking to weight the sample to match the population of Great Britain in terms of key demographics [31]. Separate weights are available for analyses of psychological distress, to account for this variable not being assessed among all participants in Wales and Scotland. All analyses used weighted data; sample sizes are reported unweighted. In an unplanned analysis, we reran the models using unweighted data.

#### Descriptive analyses

We reported descriptive data on sample characteristics for: (i) all risky drinkers; and (ii) risky drinkers who made at least one past‐year alcohol reduction attempt. Within each of these two groups, we also plotted the proportions trying to reduce their alcohol consumption for reasons including cost versus reasons not including cost within each 6‐month period across the time series. Among those who tried to reduce their alcohol consumption because of the cost, we reported the proportions who also cited additional reasons for doing so.

#### Modelled time trends

We used logistic regression to model time trends (using individual‐level data) in the prevalence of cost‐motivated alcohol reduction attempts (dependent variable) among: (i) all risky drinkers; and (ii) risky drinkers who made at least one past‐year alcohol reduction attempt, from January 2021 (around a year before the cost‐of‐living crisis began) to December 2024. We modelled survey month (independent variable) using restricted cubic splines, which allow flexible fitting of non‐linear trends over time. This method increases the precision and power of results while avoiding arbitrary categorisation or assumption of linear associations [38]. Knots represent specific points along the time axis where the behaviour of the spline can change, providing additional flexibility to the model. More knots allow for greater flexibility, but can also risk overfitting. We compared models with three, four and five knots (sufficient to accurately model trends across years without overfitting) using Akaike's information criterion (AIC). In each instance, the knots were placed at equal quantiles, which is generally considered appropriate given that the exact position of knots does not usually have a major impact on the results [38]. The best‐fitting model was selected as the model with the lowest AIC or the simplest model within two AIC units of the model with the lowest AIC (Appendix S3).

To explore moderation by level of risky drinking, nation, age, gender, occupational social grade, children in the household, smoking status and psychological distress, we repeated the models including the interaction between the moderator of interest and survey month, thus allowing for time trends to differ across subgroups. Each of the interactions was tested in a separate model with time modelled using the same number of knots as in the best‐fitting model for the overall trend. We investigated moderators on their own and not in the context of other potential confounders, as our intention was to describe rather than explain differences in trends. Level of risky drinking and age were modelled using restricted cubic splines with three knots (placed at the 5%, 50% and 95% percentiles), to allow for non‐linear relationships. We displayed estimates for specific ages (18, 25, 35, 45, 55 and 65 years) and AUDIT‐C scores (5, 8 and 12; i.e. the lowest, middle and highest possible scores within the risky drinking range) to illustrate how trends differ across ages and levels of risky drinking. Note that the models used to derive these estimates included data from participants of all ages and AUDIT‐C scores in the risky drinking range (≥5).

We used predicted estimates from our models to plot the monthly prevalence of cost‐motivated alcohol reduction attempts over the study period (overall and by moderating variables). We reported modelled estimates of prevalence in the first and last 1‐month periods in the time series and prevalence ratios (PRs) alongside 95% confidence intervals (95% CIs) calculated using bootstrapping (1000 replications).

#### Sensitivity analyses

Our primary analyses focused on monthly trends in cost‐motivated alcohol reduction attempts among all risky drinkers and among those who reported making at least one past‐year alcohol reduction attempt. In an unplanned sensitivity analysis, we restricted the primary analysis to people who only selected cost as a motive (i.e. did not also report other motives). Because our previous analysis did not include participants who responded ‘yes’ to Q2 assessing alcohol reduction attempts [28], we also reported overall trends in cost‐motivated alcohol reduction attempts excluding these participants (i.e. restricted to those responding ≥1 to Q1) for comparability.

## RESULTS

A total of 85 101 participants aged ≥18 years were surveyed in Great Britain in eligible waves between January 2021 and December 2024. We analysed data from 26 212 participants who reported risky drinking, of whom 9023 (34.4% unweighted) reported having made at least one past‐year attempt to reduce their alcohol consumption and 1355 (5.2% unweighted) reported having made at least one cost‐motivated attempt. Unweighted sample sizes within each wave are provided in Appendix S4. Weighted sample characteristics are provided in Table 1. The mean age of the participants was 45.9 years, 37.7% were women, 61.4% were in full‐time employment or self‐employed and 28.5% had children in the household. The mean AUDIT‐C score was 6.92, 21.1% reported current smoking and 30.3% reported moderate or severe past‐month psychological distress.

**TABLE 1:** Sample characteristics (aggregated across eligible waves between January 2021 and December 2024).

	Risky drinkers	Risky drinkers who made ≥1 past‐year alcohol reduction attempt
Unweighted, *n* ^a^	26 212	9023
AUDIT‐C score, mean (SD)	6.92 (1.82)	7.24 (1.88)
Nation		
England	85.6 (85.2–86.0)	87.8 (87.3–88.4)
Wales	4.8 (4.6–5.0)	4.3 (4.0–4.6)
Scotland	9.6 (9.4–9.9)	7.9 (7.4–8.3)
Age		
Mean (SD)	45.9 (17.1)	46.0 (16.0)
18–24 years	13.8 (13.3–14.3)	11.4 (10.6–12.2)
25–34 years	17.9 (17.3–18.5)	17.7 (16.8–18.7)
35–44 years	16.6 (16.1–17.2)	18.1 (17.1–19.0)
45–54 years	18.9 (18.4–19.5)	21.5 (20.6–22.5)
55–64 years	16.7 (16.2–17.2)	16.8 (16.0–17.6)
≥65 years	16.1 (15.6–16.5)	14.5 (13.8–15.3)
Missing, *n*	12	2
Gender		
Men	61.4 (60.8–62.1)	58.9 (57.8–60.1)
Women	37.7 (37.1–38.4)	40.3 (39.2–41.5)
Other	0.8 (0.7–0.9)	0.7 (0.6–0.9)
Missing, *n*	56	21
Social grade		
AB (most advantaged)	30.6 (30.0–31.3)	34.0 (32.9–35.1)
C1	30.9 (30.4–31.5)	31.6 (30.6–32.6)
C2	21.2 (20.5–21.8)	18.7 (17.8–19.8)
D	12.1 (11.5–12.6)	10.6 (9.7–11.5)
E (least advantaged)	5.2 (4.9–5.5)	5.2 (4.7–5.7)
Working status		
Full‐time employment/self‐employed	61.4 (60.7–62.0)	62.0 (60.9–63.1)
Part‐time employment	8.8 (8.4–9.2)	9.3 (8.6–10.0)
Unemployed and seeking work	2.4 (2.2–2.6)	2.7 (2.4–3.2)
Other	27.5 (26.9–28.1)	25.9 (24.9–26.9)
Missing, *n*	57	17
Children in the household		
0	71.5 (70.8–72.1)	71.2 (70.1–72.2)
1	12.9 (12.4–13.4)	12.8 (12.1–13.7)
≥2	15.6 (15.1–16.1)	16.0 (15.1–16.9)
Smoking status		
Never	48.8 (48.1–49.5)	48.6 (47.4–49.8)
Former	30.0 (29.4–30.6)	33.1 (32.0–34.2)
Current	21.1 (20.6–21.7)	18.3 (17.4–19.3)
Missing, *n*	173	64
Past‐month psychological distress^b^		
No/low	69.6 (68.8–70.4)	63.1 (61.7–64.5)
Moderate	23.9 (23.2–24.7)	28.3 (27.0–29.6)
Severe	6.4 (6.0–6.9)	8.6 (7.8–9.5)
Missing, *n* ^b^	8841	2892

*Note*: Risky drinkers are defined as those scoring ≥5 on the Alcohol Use Disorders Identification Test – Consumption (AUDIT‐C) scale.

Data are column percentages with 95% confidence intervals, unless otherwise specified. There were missing data on some variables; valid percentages are shown.

^a^ Unweighted sample sizes by year: risky drinkers in 2021 *n* = 8805, 2022 *n* = 5220, 2023 *n* = 6570 and 2024 *n* = 5617; risky drinkers who made ≥1 past‐year alcohol reduction attempts in 2021 *n* = 3039, 2022 *n* = 1738, 2023 *n* = 2262 and 2024 *n* = 1984.

^b^ Data on psychological distress were collected from approximately 50% of participants in Wales and Scotland and were not collected at all after June 2023; missing cases include those not asked the relevant questions or who were surveyed in waves in which distress was not assessed.

### Overall trends

**FIGURE 1:**
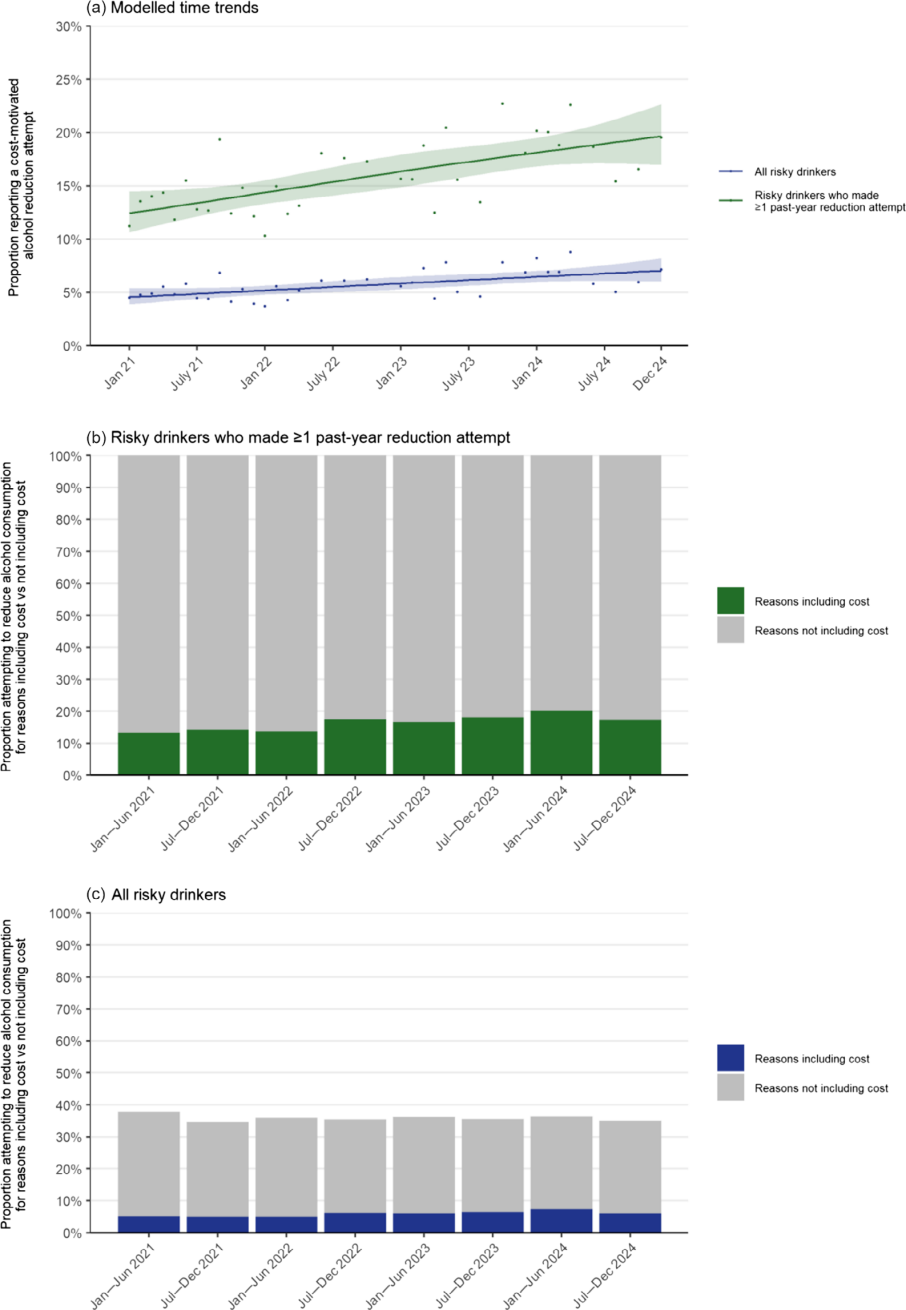
Prevalence of cost‐motivated alcohol reduction attempts among risky drinkers (aged ≥18 years) in Great Britain, from January 2021 to December 2024. Risky drinkers are defined as those scoring ≥5 on the Alcohol Use Disorders Identification Test – Consumption (AUDIT‐C) scale. Panel (a) shows the modelled monthly time trends. Lines represent the modelled weighted proportion reporting cost‐motivated alcohol reduction attempts by monthly survey wave, modelled non‐linearly using restricted cubic splines (three knots; for model selection, see Appendix S3). Shaded bands represent 95% confidence intervals. Points represent the unmodelled weighted proportion by month. Note that participants could select multiple motives for reduction attempts, so in panel (a) cost might not be the sole reason for the reduction attempts. Panels (b) and (c) show weighted data aggregated across 6‐month periods, among all risky drinkers and among risky drinkers who attempted to reduce their consumption in the past year, respectively. Bars represent the proportions trying to reduce their alcohol consumption for reasons including cost versus not including cost. Corresponding estimates for all risky drinkers are provided in Appendix S5. Corresponding estimates using more detailed (pre‐registered) categorisations for nation, social grade, working status, children in the household and psychological distress are provided in Appendix S1.

Between January 2021 and December 2024, the proportion of risky drinkers in Great Britain who reported making a cost‐motivated alcohol reduction attempt in the past year increased from 4.6% to 7.0% (PR = 1.54, 95% CI = 1.34–1.74; Figure 1a, with further details in Appendix S5). This reflected cost becoming an increasingly prevalent motive among those trying to reduce their alcohol consumption (Figure 1b), rising from 12.4% to 19.7% (PR = 1.58, 95% CI = 1.39–1.77; Figure 1a; Table 2). The overall proportion of risky drinkers trying to reduce their consumption (either for cost or other reasons) was relatively stable across the period (at an average of 36.0%; Figure 1c).

**TABLE 2:** Modelled estimates of changes in the prevalence of cost‐motivated alcohol reduction attempts from January 2021 to December 2024 among risky drinkers who made ≥1 past‐year alcohol reduction attempts.

	Prevalence, % (95% CI)^a^		Prevalence ratio (95% CI)^b^
	Jan 2021	Dec 2024	
Overall	12.4 (10.7–14.5)	19.7 (17.0–22.7)	1.58 (1.39–1.77)
Level of risky drinking (AUDIT‐C score)^c^			
5 (lowest)	11.1 (8.1–15.2)	17.6 (12.9–23.6)	1.58 (0.96–2.20)
8	12.0 (9.5–15.0)	20.5 (16.8–24.8)	1.71 (1.26–2.16)
12 (highest)	21.7 (13.5–33.1)	22.6 (13.2–36.1)	1.04 (0.37–1.71)
Nation			
England	12.5 (10.7–14.7)	19.7 (16.9–22.9)	1.57 (1.27–1.88)
Wales/Scotland	10.3 (6.2–16.5)	18.0 (11.4–27.2)	1.75 (0.66–2.84)
Age^d^			
18 years	32.5 (23.9–42.5)	39.0 (28.8–50.3)	1.20 (0.77–1.64)
25 years	23.1 (18.3–28.7)	32.1 (25.7–39.1)	1.39 (1.01–1.77)
35 years	13.9 (11.5–16.8)	23.6 (19.7–28.1)	1.70 (1.31–2.09)
45 years	9.2 (7.3–11.7)	17.6 (13.9–22.0)	1.90 (1.35–2.46)
55 years	7.8 (6.2–9.8)	13.8 (11.0–17.2)	1.77 (1.27–2.28)
65 years	8.3 (6.4–10.9)	11.5 (8.9–14.7)	1.38 (0.92–1.84)
Gender			
Men	12.1 (9.8–14.9)	18.9 (15.5–22.8)	1.56 (1.17–1.95)
Women	12.8 (10.1–15.9)	20.6 (16.3–25.7)	1.61 (1.17–2.07)
Social grade			
ABC1 (most advantaged)	10.4 (8.7–12.4)	19.1 (16.2–22.4)	1.84 (1.45–2.22)
C2DE (least advantaged)	16.8 (12.9–21.6)	21.2 (16.1–27.5)	1.27 (0.86–1.68)
Working status			
Full‐time employment/self‐employed	10.3 (8.3–12.7)	19.3 (16.0–23.1)	1.88 (1.40–2.35)
Part‐time employment/unemployed and seeking work/other	15.8 (12.7–19.6)	20.0 (15.7–25.2)	1.26 (0.91–1.62)
Children in the household			
0	12.7 (10.6–15.2)	19.0 (16.0–22.5)	1.50 (1.16–1.83)
≥1	11.9 (8.9–15.7)	21.3 (16.1–27.6)	1.79 (1.15–2.44)
Smoking status			
Never	11.8 (9.5–14.7)	18.2 (14.6–22.4)	1.54 (1.11–1.96)
Former	10.4 (7.8–13.7)	19.7 (15.4–24.9)	1.89 (1.28–2.50)
Current	19.9 (14.3–26.9)	23.5 (16.7–32.0)	1.18 (0.70–1.66)
Past‐month psychological distress^e^			
No/low	8.9 (7.0–11.3)	11.2 (6.5–18.6)	1.26 (0.81–1.71)
Moderate/severe	19.2 (15.3–23.8)	40.5 (27.1–55.4)	2.11 (1.76–2.46)

*Note*: Risky drinkers are defined as those scoring ≥5 on the Alcohol Use Disorders Identification Test – Consumption (AUDIT‐C) scale.

^a^ Data are weighted estimates of prevalence in the first and last months of the study period from logistic regression with survey month modelled non‐linearly using restricted cubic splines (three knots; for model selection, see Appendix S3).

^b^ Prevalence ratio calculated as prevalence in December 2024 (or June 2023, for estimates by history of mental health conditions) divided by prevalence in January 2021 with 95% CIs calculated using bootstrapping (1000 replications).

^c^ AUDIT‐C scores for risky drinkers range from 5 to 12. Modelled estimates are shown for selected scores to illustrate differences. Note that the model used to derive these estimates included data from participants with any score on this scale, not only those with a score of exactly 5, 8 or 12.

^d^ Modelled estimates are shown for selected ages to illustrate differences. Note that the model used to derive these estimates included data from participants of all ages, not only those who were aged exactly 18, 25, 35, 45, 55 or 65 years.

^e^ Data on psychological distress were not collected after June 2023; estimates shown are therefore for January 2021 and June 2023, rather than January 2021 and December 2024.

The pattern of the results was similar in the unweighted analyses (Appendix S6) and in the analyses where we restricted the outcome to alcohol reduction attempts *only* motivated by cost (Appendix S7). This represented just 17.3% (95% CI = 15.0%–19.7%) of all cost‐motivated alcohol reduction attempts. The most commonly cited motives alongside cost were improving fitness (56.6%), a concern about future health problems (51.4%) and weight loss (50.0%) (Appendix S7).

### Trends within population subgroups

**FIGURE 2:**
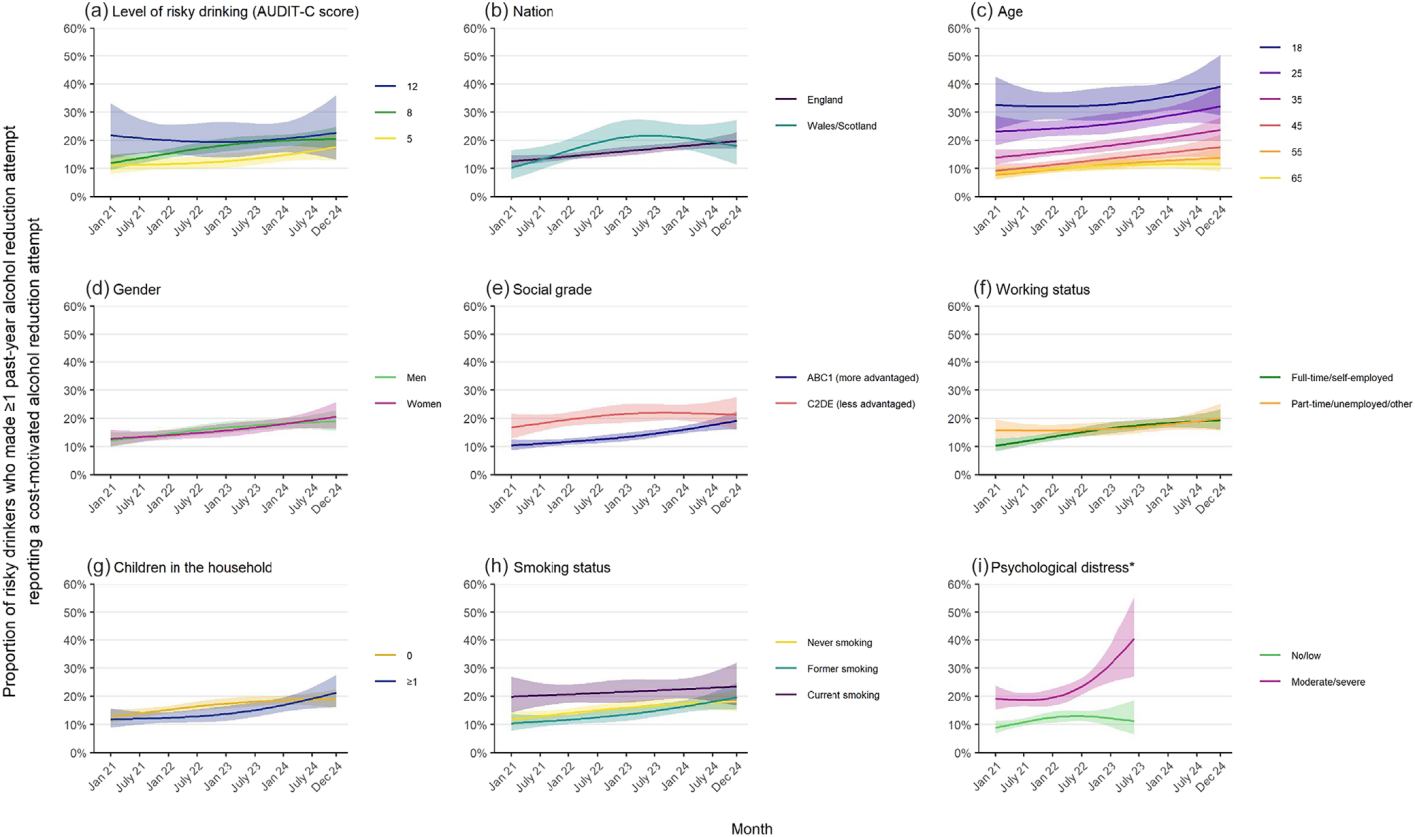
Trends in the monthly prevalence of cost‐motivated alcohol reduction attempts among subgroups of risky drinkers (aged ≥18 years) in Great Britain who made one or more past‐year alcohol reduction attempts, from January 2021 to December 2024. Risky drinkers are defined as those scoring ≥5 on the Alcohol Use Disorders Identification Test – Consumption (AUDIT‐C) scale. Lines represent the modelled weighted proportion reporting cost‐motivated alcohol reduction attempts by monthly survey wave (modelled non‐linearly using restricted cubic splines with three knots) and (a) level of risky drinking, (b) nation, (c) age, (d) gender, (e) social grade, (f) working status, (g) children in the household, (h) smoking status and (i) psychological distress. Shaded bands represent 95% confidence intervals. Points represent the unmodelled weighted proportion by month. *Data on psychological distress were only available up to June 2023. Corresponding figures for all risky drinkers are provided in Appendix S5. Corresponding figures using unweighted data are provided in Appendix S6. Corresponding figures using more detailed (pre‐registered) categorisations for nation, social grade, working status, children in the household and psychological distress are provided in Appendix S1.

Increases in the monthly prevalence of cost‐motivated alcohol reduction attempts were observed across most subgroups. Here, we focus on results from risky drinkers who reported one or more past‐year alcohol reduction attempts (Figure 2; Table 2). The pattern of results was very similar among all risky drinkers (Apendix S5) and among the restricted sample excluding those who only reported that they were currently trying to cut down (i.e. excluding those who did not report ≥1 in response to Q1; Appendix S7). Results were also similar when we analysed unweighted data, with the exception of trends by psychological distress (as described below; Appendix S6).

The monthly prevalence of cost‐motivated alcohol reduction attempts was higher overall across the study period among those with higher AUDIT‐C scores. The increase was similar among those with lower and intermediate levels of risky drinking but was absent among the heaviest drinkers (AUDIT‐C = 12; PR = 0.96, 95% CI = 0.51–1.78), to the extent that the prevalence was similar among all these groups by the end of the period (Figure 2a; Table 2).

The monthly prevalence of cost‐motivated alcohol reduction attempts in England was generally similar to that in Wales/Scotland. While the overall change across the period was similar, the increase appeared to be more linear in England, with a slightly more rapid increase in Wales/Scotland in 2021–2022, before levelling off in 2023 and potentially starting to decline (Figure 2b; Table 2). Prevalence was consistently higher across the period at younger ages, with quite large differences across the age spectrum, but the increase over time was broadly similar (Figure 2c; Table 2). There were no notable differences by gender (Figure 2d; Table 2) or by presence of children in the household (Figure 2g; Table 2).

The monthly prevalence of cost‐motivated alcohol reduction attempts was higher at the start of the period among those from less versus more advantaged social grades (16.8% vs 10.4%). However, there was an uncertain greater increase over time among the more advantaged group (PR = 1.84, 95% CI = 1.47–2.27, vs PR = 1.27, 95% CI = 0.91–1.72; Table 2), particularly in the latter half of the period (Figure 2e), which meant that by the end of 2024, prevalence was more similar in both groups (Table 2). A similar pattern was observed by working status and smoking status. Those in full‐time employment or who were self‐employed had lower prevalence initially but an uncertain greater increase over time than those who were part‐time employed, unemployed and seeking work, or who had other working status (Figure 2f; Table 2). Those who reported former or never smoking had lower prevalence initially but an uncertain greater increase over time than those who reported current smoking (Figure 2h; Table 2).

The monthly prevalence of cost‐motivated alcohol reduction attempts was consistently higher across the period among those experiencing moderate/severe psychological distress than those reporting no/low distress. The weighted model suggested the increase in cost‐motivated alcohol reduction attempts was largely concentrated among those experiencing moderate/severe distress, from 19.2% to 40.5% (PR = 2.11; 95% CI = 1.76–2.46), with an uncertain increase among those experiencing no/low distress (from 8.5% to 11.2%; PR = 1.26; 95% CI = 0.81–1.71; Figure 2I; Table 2). However, the unweighted model suggested a more modest, uncertain increase among those experiencing moderate/severe distress (from 18.7% to 21.9%; PR = 1.17, 95% CI = 0.85–1.48; Appendix S6).

## DISCUSSION

Between 2021 and 2024, there was a notable rise in the monthly prevalence of cost‐motivated alcohol reduction attempts among risky drinkers in Great Britain. This was driven by an increase in the proportion of attempts that were motivated by the cost of drinking as opposed to an increase in overall alcohol reduction attempts. In January 2021, around one in eight risky drinkers who had tried to reduce their alcohol consumption in the past year said they did so because drinking was too expensive. By December 2024, this number had risen to one in five. This equates to approximately 1.1 million people making a cost‐motivated alcohol reduction attempt in 2024 (52.7 million adults aged ≥18 years in Great Britain [39] multiplied by 30% reporting risky drinking [7] multiplied by 7.0% reporting a past‐year cost‐motivated alcohol reduction attempt). Most people who reported trying to reduce their alcohol consumption because of cost also cited other motives, such as health concerns.

Several factors likely contributed to this increase. There has been a cost‐of‐living crisis in Great Britain since late 2021 [19], which has put considerable pressure on household budgets [20, 21]. In addition, reforms to the alcohol duty system and, in particular, higher alcohol taxes were implemented in August 2023. We had anticipated that we might see more people trying to reduce their alcohol consumption to save money in response to these changes. Our results show a linear increase in cost‐motivated attempts across the study period, suggesting this was a gradual change rather than an abrupt shift. This perhaps reflects the incremental nature of inflation, which raises prices progressively over time, and the role of rising interest rates, which impact housing costs over time as homeowner fixed‐rate mortgage deals expire, and the erosion of household savings. The linear increase also pre‐dated the cost‐of‐living crisis and duty reforms, suggesting that other factors, such as the economic impacts of the COVID‐19 pandemic (which caused job losses or reduced income for many people [40]) or the anticipated effects of the duty reforms being implemented (as has been observed previously for tobacco control policies [41]) may also have played a role.

At the start of the study period, cost‐motivated alcohol reduction attempts were more prevalent among subgroups of risky drinkers who typically have less money to spend and have been identified as being more likely to experience financial hardship during the cost‐of‐living crisis [21]. These included those who were younger, those from less advantaged occupational social grades, those not in full‐time employment (or who were self‐employed), those who reported current smoking and those experiencing moderate to severe psychological distress. Prevalence was also higher among those who drank more heavily; on average, this group spend more on alcohol (in total, as opposed to per unit) compared with those who drink less heavily [42]. Experiencing financial hardship and spending more on alcohol are both likely to make cost a more salient motive for trying to reduce consumption, especially in the context of the cost‐of‐living crisis and increasing alcohol prices.

Over time, the general upward trend in cost‐motivated alcohol reduction attempts was observed in almost all subgroups. Geographically, the overall increase was similar across the three nations but appeared to occur more rapidly in Wales and Scotland than in England. It is possible that minimum unit pricing policies in Wales and Scotland, which limit the extent to which people can switch their purchasing to cheaper products, prompted drinkers in these nations to reduce their consumption at an earlier stage of the cost‐of‐living crisis than those in England (where there is no minimum unit price for alcohol) [43]. There may also have been some anticipatory changes in advance of the reforms to the alcohol duty system being implemented in Wales and Scotland because people in these countries had recent experience of how legislative changes (i.e. minimum unit pricing) can affect alcohol prices.

Trends were similar across ages, for men and women, and for those with and without children in the household. However, increases in cost‐motivated alcohol reduction attempts were greater among those from more advantaged social grades and those in full‐time employment, causing existing differences to narrow. This may reflect cost already being a more important motive among less advantaged groups at baseline, prior to the cost‐of‐living crisis and duty reforms, but becoming a more relevant consideration for more advantaged groups as the period of economic hardship continued (e.g. as housing costs began to increase). In a previous analysis using data up to December 2022, we found cost‐motivated alcohol reduction attempts increased only among those from less advantaged social grades [28]. However, the present analysis, over a longer period, also shows a later increase among those who were more advantaged. Although the cost‐of‐living crisis has put significant financial strain on households since late 2021, many working‐class families have struggled with household budgets for much longer than this [44]. It is also possible that more advantaged groups started to cite cost as a reason for trying to reduce their alcohol consumption because the price of goods like alcohol was prominent in public debate, even if they were not actually experiencing a significant squeezing of their finances (i.e. they did not need to cut down because of cost, they just cited it as a relevant factor). This would help to explain why we did not observe an increase in the overall prevalence of reduction attempts. However, in an unplanned analysis of attempts only motivated by cost the pattern of results was similar.

Trends also differed by smoking status and level of risky drinking. Increases over time were greater among those who reported former or never smoking and among those reporting lower levels of risky drinking—groups that were initially less likely to report trying to reduce their consumption for cost reasons. However, those who currently smoked initially had the highest prevalence of attempts to cut down drinking, but these attempts increased by less across the period of the study, suggesting that the increase in price had minimal impact on risky drinkers who smoked attempting to reduce their alcohol consumption. It is likely that the smaller increase among people who smoked was confounded by social grade, as smoking is much more common among socio‐economically disadvantaged groups [45]. The smaller increase among heavier risky drinkers may reflect their lower sensitivity to price [46]; they may have already adapted to higher expenditure by purchasing cheaper alcohol or in bulk, while other motivations, such as health concerns or social pressures remained more influential over time.

In contrast to the patterns observed by socio‐economic indicators, smoking, and level of risky drinking, the increase in cost‐motivated alcohol reduction attempts was greater among those experiencing moderate to severe psychological distress, despite this group having a higher prevalence of such attempts at baseline. This may be partly explained by changes in the demographic profile of those experiencing distress over the study period; for example, recent data show increases in distress have been particularly pronounced among younger adults [47], who we found tended to be more likely to report cost‐motivated alcohol reduction attempts.

This study had several strengths. These include the large, representative sample and the detailed assessment of socio‐demographic characteristics. In addition, the survey pre‐dated the cost‐of‐living crisis, and the monthly data collection permitted detailed analysis of trends. However, there were also limitations. First, all data were self‐reported and questions about alcohol reduction attempts relied on recall of the past year. This may introduce recall bias, but we would expect any such bias to be relatively consistent across the time series so this would not explain the changes that we observed over time. Second, only those who reported risky drinking were asked about past‐year alcohol reduction attempts, meaning those who had successfully cut down and reduced their AUDIT‐C score below the threshold were not included in this analysis. This may have affected our results if the success of alcohol reduction attempts changed as financial pressures increased. Future studies should examine the success of cost‐motivated reduction attempts relative to other attempts and also the extent to which purchasing has changed during recent years, as people may not recognise all reductions in alcohol consumption or describe them as ‘attempts’. Third, data on psychological distress were not collected across the entire period, limiting the number of time points for this analysis. In addition, the pattern of trends by distress differed somewhat between weighted and unweighted analyses, introducing some uncertainty. Finally, the observational study design means that causality cannot be established. While we have speculated on potential explanations for our findings, further research (e.g. qualitative) is needed to explore the underlying mechanisms driving increases in cost‐motivated alcohol reduction attempts and differences between population subgroups.

In conclusion, cost is an increasingly important motive for alcohol reduction attempts among risky drinkers in Great Britain, likely reflecting financial pressures resulting from the cost‐of‐living crisis and rising alcohol prices. There is prior evidence that economic downturns (usually defined as a recession rather than high inflation) are associated with reductions in deaths from alcohol [48, 49]. However, increases in deaths that began during the pandemic have continued during our study period [4, 5]. Our results suggest a partial explanation for this as we find that cost is playing a greater role in reduction attempts but that the prevalence of reduction attempts is not increasing. Given recent evidence that suggests attempts motivated by other reasons (e.g. health concerns, social factors or health professional advice) have not decreased [50], it seems that financial pressures have provided added motivation to reduce consumption rather than displacing other motives or increasing the rate of reduction attempts. Our findings suggest that alcohol support services should be aware that an increasing proportion of risky drinkers who attempt to cut down are increasingly motivated by cost and could consider also providing more tailored support for financial hardship.

## AUTHOR CONTRIBUTIONS

**Sarah E. Jackson:** Conceptualization (equal); data curation (equal); formal analysis (lead); funding acquisition (supporting); investigation (equal); methodology (equal); visualization (lead); writing—original draft (lead); writing—review and editing (equal). **Jamie Brown:** Conceptualization (equal); data curation (equal); funding acquisition (equal); investigation (equal); methodology (equal); writing—review and editing (equal). **Colin Angus:** Conceptualization (equal); funding acquisition (equal); investigation (equal); methodology (equal); writing—review and editing (equal). **Abi Stevely:** Funding acquisition (supporting); investigation (supporting); methodology (supporting); writing—review and editing (equal). **Magdalena Opazo Breton:** Investigation (supporting); methodology (supporting); writing—review and editing (equal). **Leonie Brose:** Investigation (supporting); methodology (supporting); writing—review and editing (equal). **Luke Wilson:** Investigation (supporting); methodology (supporting); writing—review and editing (equal). **John Holmes:** Conceptualization (equal); funding acquisition (equal); investigation (equal); methodology (equal); writing—review and editing (equal).

## DECLARATION OF INTERESTS

J.B. has received unrestricted research funding from Pfizer and J&J (most recently in 2018), who manufacture smoking cessation medications. All authors declare no financial links with the alcohol or tobacco industry, or their representatives.

## Supporting information


**Appendix S1.** Analyses using pre‐registered categorisations.

**Appendix S2.** Comparison of participants with versus without missing data on individual characteristics.

**Appendix S3.** Model selection.

**Appendix S4.** Unweighted sample sizes by wave.

**Appendix S5.** Primary analyses for all risky drinkers.

**Appendix S6.** Unweighted analyses.

**Appendix S7.** Sensitivity analyses restricting the definition of reduction attempts.

## Data Availability

The data used in these analyses are available on the Open Science Framework (https://osf.io/cp5d7/), with age provided in bands to preserve anonymity. The statistical code is available from the corresponding author, on request.
