# Supplementary material for: Examining changes in the prevalence of cost‐motivated alcohol reduction attempts in the context of a cost‐of‐living crisis and alcohol duty reforms: A population survey of risky drinkers in Great Britain, 2021–2024
Source: Addiction. 2025 Nov 19;121(4):825–38. doi: 10.1111/add.70248 (PMC12980291; doi:10.1111/add.70248)
Supplement: Supplementary file 1 — Appendix S1. Analyses using pre‐registered categorisations. [file ADD-121-825-s002.docx]

## Contents

Table 1: Modelled estimates, risky drinkers who made ≥1 past-year alcohol reduction attempt

Figure 1: Trends, subgroups of risky drinkers who made ≥1 past-year alcohol reduction attempt

Table 2: Modelled estimates, risky drinkers

Figure 2: Trends, subgroups of risky drinkers

## Table 1. Modelled estimates of changes in the prevalence of cost-motivated alcohol reduction attempts from January 2021 to December 2024 among risky drinkers who made ≥1 past-year alcohol reduction attempt – pre-registered categorisations

|  | **Prevalence, % [95%CI]^1^** | | **Prevalence ratio [95%CI]^2^** |
| --- | --- | --- | --- |
|  | **Jan 2021** | **Dec 2024** |  |
|  |  |  |  |
| Nation |  |  |  |
| England | 12.5 [10.5–14.8] | 19.9 [16.9–23.3] | 1.60 [1.28-1.92] |
| Wales | 10.3 [6.2–16.5] | 18.0 [11.4–27.2] | 1.75 [0.65-2.85] |
| Scotland | 13.1 [9.2–18.4] | 17.3 [11.8–24.7] | 1.32 [0.71-1.93] |
|  |  |  |  |
| Social grade |  |  |  |
| AB (most advantaged) | 8.1 [6.0–11.0] | 16.2 [12.4–20.9] | 1.99 [1.23-2.74] |
| C1 | 13 [10.4–16.0] | 22.5 [18.3–27.3] | 1.73 [1.27-2.19] |
| C2 | 14.6 [9.9–21.0] | 23.1 [16.5–31.5] | 1.58 [0.80-2.37] |
| D | 18.8 [11.1–30.1] | 18.3 [8.9–34.1] | 0.97 [0.23-1.72] |
| E (least advantaged) | 19.7 [12.4–29.8] | 19.7 [11.3–32.0] | 1.00 [0.34-1.66] |
|  |  |  |  |
| Working status |  |  |  |
| Full-time employment/self-employed | 10.3 [8.3–12.7] | 19.3 [16.0–23.1] | 1.88 [1.41-2.34] |
| Part-time employment | 16.8 [10.4–26.1] | 27.6 [16.5–42.4] | 1.64 [0.60-2.68] |
| Unemployed and seeking work | 14.5 [5.7–32.2] | 19.2 [8.0–39.4] | 1.33 [0.00-3.45] |
| Other | 15.9 [12.4–20.3] | 17.9 [13.3–23.7] | 1.12 [0.75-1.50] |
|  |  |  |  |
| Children in the household |  |  |  |
| 0 | 12.7 [10.6–15.2] | 19.0 [16.0–22.5] | 1.50 [1.16-1.83] |
| 1 | 13.5 [8.8–20.1] | 23.4 [16.0–32.9] | 1.74 [0.79-2.68] |
| ≥2 | 10.8 [7.2–15.8] | 19.4 [12.7–28.5] | 1.80 [0.84-2.77] |
|  |  |  |  |
| Past-month psychological distress^3^ |  |  |  |
| No/low | 8.9 [7.0–11.3] | 11.2 [6.5–18.6] | 1.26 [0.79-1.72] |
| Moderate | 18.3 [14.1–23.3] | 37.1 [22.9–54.0] | 2.03 [1.61-2.45] |
| Severe | 23.4 [14.1–36.1] | 50.5 [22.9–77.9] | 2.16 [1.36-2.97] |
|  |  |  |  |

^1^ Data are weighted estimates of prevalence in the first and last months in the study period from logistic regression with survey month modelled non-linearly using restricted cubic splines (three knots; see **Supplementary File 2** for model selection).

^2^ Prevalence ratio calculated as prevalence in December 2024 (or June 2023, for estimates by history of mental health conditions) divided by prevalence in January 2021 with 95% CIs calculated using bootstrapping (1,000 replications).

^3^ Data on psychological distress were not collected after June 2023; estimates shown are therefore for January 2021 and June 2023, rather than January 2021 and December 2024.


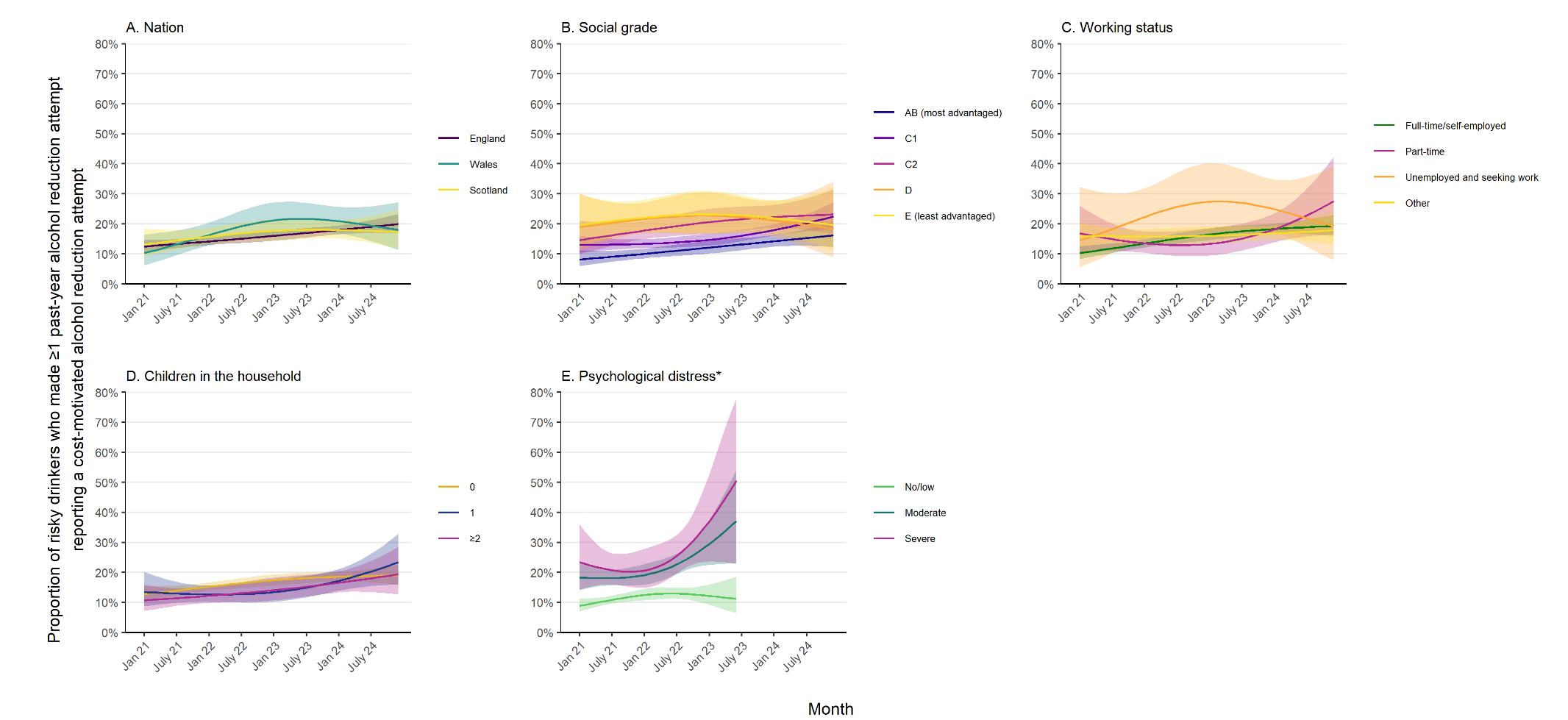


## Figure 1. Trends in the prevalence of cost-motivated alcohol reduction attempts among subgroups of risky drinkers (≥18y) in Great Britain who made ≥1 past-year alcohol reduction attempt, January 2021 to December 2024 – pre-registered categorisations. Lines represent the modelled weighted proportion reporting cost-motivated alcohol reduction attempts by monthly survey wave (modelled non-linearly using restricted cubic splines with three knots) and (A) nation, (B) social grade, (C) working status, (D) children in the household, and (E) psychological distress. Shaded bands represent 95% confidence intervals. Points represent the unmodelled weighted proportion by month. *Data on psychological distress were only available up to June 2023.

## Table 2. Modelled estimates of changes in the prevalence of cost-motivated alcohol reduction attempts from January 2021 to December 2024 among risky drinkers – pre-registered categorisations

|  | **Prevalence, % [95%CI]^1^** | | **Prevalence ratio [95%CI]^2^** |
| --- | --- | --- | --- |
|  | **Jan 2021** | **Dec 2024** |  |
|  |  |  |  |
| Nation |  |  |  |
| England | 4.7 [3.9–5.6] | 7.3 [6.1–8.6] | 1.55 [1.22-1.89] |
| Wales | 3.8 [2.3–6.2] | 6.2 [3.9–9.9] | 1.66 [0.65-2.66] |
| Scotland | 3.8 [2.6–5.6] | 5.0 [3.4–7.3] | 1.30 [0.64-1.96] |
|  |  |  |  |
| Social grade |  |  |  |
| AB (most advantaged) | 3.4 [2.5–4.6] | 6.7 [5.1–8.8] | 1.97 [1.15-2.80] |
| C1 | 5..0 [4.0–6.3] | 8.2 [6.6–10.2] | 1.64 [1.19-2.10] |
| C2 | 4.4 [3.0–6.6] | 7.1 [4.9–10.1] | 1.59 [0.78-2.41] |
| D | 5.4 [3.1–9.3] | 4.8 [2.2–10.1] | 0.89 [0.15-1.64] |
| E (least advantaged) | 7.2 [4.4–11.5] | 7.7 [4.5–13.0] | 1.08 [0.27-1.89] |
|  |  |  |  |
| Working status |  |  |  |
| Full-time employment/self-employed | 3.7 [2.9–4.6] | 7.0 [5.8–8.5] | 1.91 [1.40-2.41] |
| Part-time employment | 6.5 [3.9–10.5] | 9.4 [5.4–15.9] | 1.45 [0.39-2.51] |
| Unemployed and seeking work | 4.8 [1.8–12.5] | 8.4 [3.7–17.8] | 1.74 [0.00-4.64] |
| Other | 6.0 [4.6–7.8] | 6.1 [4.5–8.4] | 1.02 [0.64-1.40] |
|  |  |  |  |
| Children in the household |  |  |  |
| 0 | 4.6 [3.8–5.6] | 6.9 [5.7–8.2] | 1.49 [1.15-1.84] |
| 1 | 4.9 [3.1–7.5] | 8.5 [5.7–12.5] | 1.75 [0.71-2.79] |
| ≥2 | 4.2 [2.8–6.4] | 6.6 [4.2–10.2] | 1.57 [0.62-2.51] |
|  |  |  |  |
| Past-month psychological distress^3^ |  |  |  |
| No/low | 3.1 [2.4–3.9] | 4.0 [2.3–6.8] | 1.28 [0.82-1.75] |
| Moderate | 8.0 [6.1–10.5] | 19.1 [11.1–30.8] | 2.37 [1.92-2.82] |
| Severe | 10.7 [6.4–17.2] | 24.2 [9.5–49.4] | 2.27 [1.39-3.15] |
|  |  |  |  |

^1^ Data are weighted estimates of prevalence in the first and last months in the study period from logistic regression with survey month modelled non-linearly using restricted cubic splines (three knots; see **Supplementary File 2** for model selection).

^2^ Prevalence ratio calculated as prevalence in December 2024 (or June 2023, for estimates by history of mental health conditions) divided by prevalence in January 2021 with 95% CIs calculated using bootstrapping (1,000 replications).

^3^ Data on psychological distress were not collected after June 2023; estimates shown are therefore for January 2021 and June 2023, rather than January 2021 and December 2024.


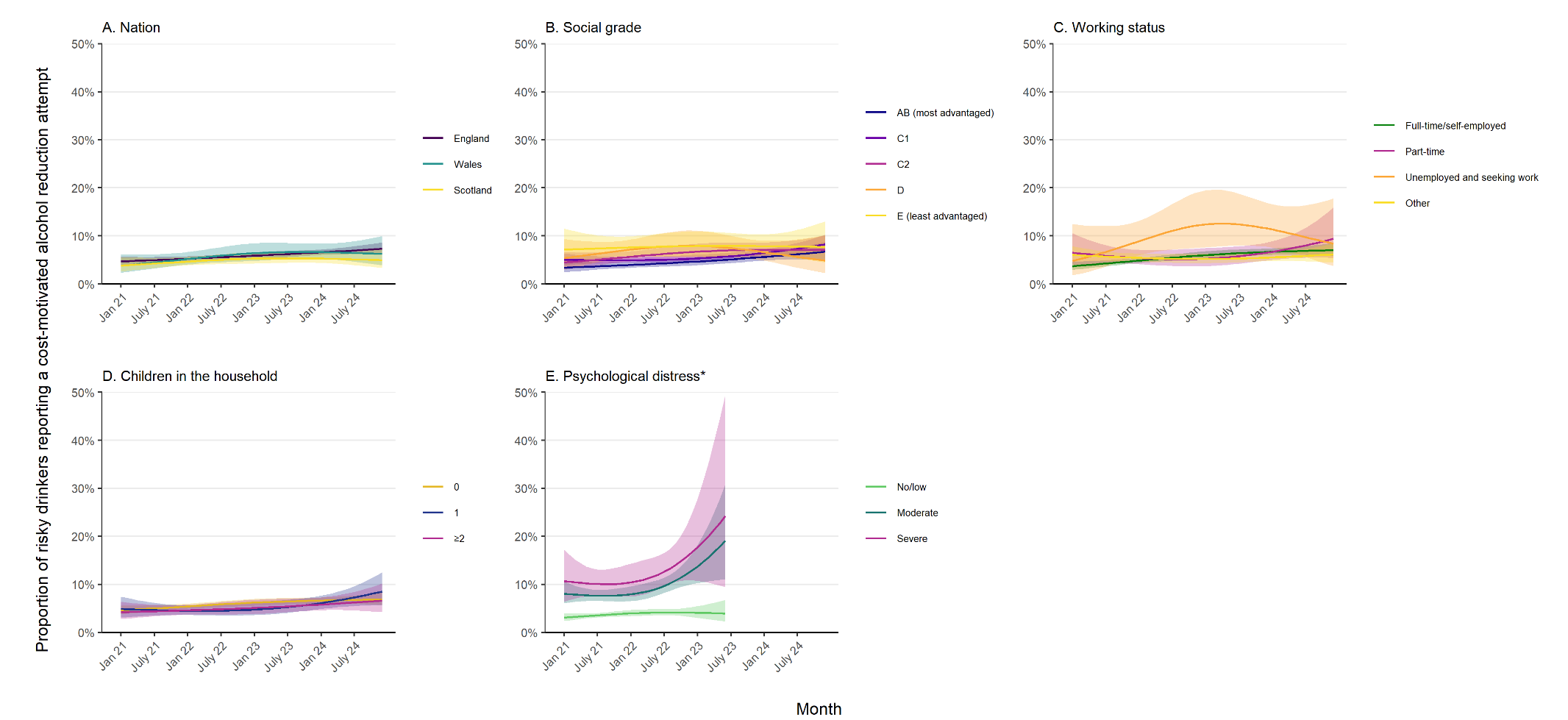


## Figure 2. Trends in the prevalence of cost-motivated alcohol reduction attempts among subgroups of risky drinkers (≥18y) in Great Britain, January 2021 to December 2024 – pre-registered categorisations. Lines represent the modelled weighted proportion reporting cost-motivated alcohol reduction attempts by monthly survey wave (modelled non-linearly using restricted cubic splines with three knots) and (A) nation, (B) social grade, (C) working status, (D) children in the household, and (E) psychological distress. Shaded bands represent 95% confidence intervals. Points represent the unmodelled weighted proportion by month. *Data on psychological distress were only available up to June 2023.
