# Supplementary material for: Examining changes in the prevalence of cost‐motivated alcohol reduction attempts in the context of a cost‐of‐living crisis and alcohol duty reforms: A population survey of risky drinkers in Great Britain, 2021–2024
Source: Addiction. 2025 Nov 19;121(4):825–38. doi: 10.1111/add.70248 (PMC12980291; doi:10.1111/add.70248)
Supplement: Supplementary file 2 — Appendix S2. Comparison of participants with versus without missing data on individual characteristics. [file ADD-121-825-s003.docx]

**Contents**

Table 1. Alcohol reduction attempts among those with vs. without missing data

**Table 1.** Prevalence of past-year alcohol reduction attempts among risky drinkers with vs. without missing data on individual characteristics

|  | **Made ≥1 past-year alcohol reduction attempt** | **Made ≥1 cost-motivated past-year alcohol reduction attempts** |
| --- | --- | --- |
|  |  |  |
| Age |  |  |
| Provided data | 36.0 [35.3-36.6] | 5.7 [5.4-6.1] |
| Missing | 20.0 [0.0-44.8] | 0 [0-0] |
|  |  |  |
| Gender |  |  |
| Provided data | 36.0 [35.3-36.6] | 5.7 [5.4-6.1] |
| Missing | 38.2 [25.3-51.0] | 5.5 [0.0-11.5] |
|  |  |  |
| Working status |  |  |
| Provided data | 36.0 [35.3-36.7] | 5.7 [5.4-6.1] |
| Missing | 30.8 [17.1-44.5] | 4.0 [0.0-8.3] |
|  |  |  |
| Smoking status |  |  |
| Provided data | 35.9 [35.2-36.6] | 5.7 [5.4-6.1] |
| Missing | 42.8 [33.7-51.8] | 6.6 [2.0-11.2] |
|  |  |  |
| Past-month psychological distress^2^ |  |  |
| Provided data | 36.6 [35.7-37.4] | 5.5 [5.1-5.9] |
| Missing | 34.5 [33.3-35.7] | 6.3 [5.7-7.0] |
|  |  |  |

Note: There were no missing data on nation, social grade, or children in the household so these variables are not presented in the table. Risky drinking was defined according to AUDIT-C scores so data could not be provided for participants missing data on AUDIT-C.
