# Supplementary material for: Examining changes in the prevalence of cost‐motivated alcohol reduction attempts in the context of a cost‐of‐living crisis and alcohol duty reforms: A population survey of risky drinkers in Great Britain, 2021–2024
Source: Addiction. 2025 Nov 19;121(4):825–38. doi: 10.1111/add.70248 (PMC12980291; doi:10.1111/add.70248)
Supplement: Supplementary file 3 — Appendix S3. Model selection. [file ADD-121-825-s007.docx]

## Contents

Table 1: Model selection

## Table 1. Model selection: AIC values for models with 3, 4, and 5 knots

|  | **AIC** | | |
| --- | --- | --- | --- |
|  | **3 knots** | **4 knots** | **5 knots** |
|  |  |  |  |
| Trends among… |  |  |  |
| All risky drinkers | 11158.61 | 11158.29 | 11161.03 |
| Risky drinkers who made ≥1 past-year alcohol reduction attempt | 7882.76 | 7884.05 | 7886.49 |
|  |  |  |  |

AIC, Akaike Information Criterion.

Shaded cells indicate the best fitting model (the model with the lowest AIC or the simplest model within 2 AIC units)
