# Supplementary material for: Examining changes in the prevalence of cost‐motivated alcohol reduction attempts in the context of a cost‐of‐living crisis and alcohol duty reforms: A population survey of risky drinkers in Great Britain, 2021–2024
Source: Addiction. 2025 Nov 19;121(4):825–38. doi: 10.1111/add.70248 (PMC12980291; doi:10.1111/add.70248)
Supplement: Supplementary file 4 — Appendix S4. Unweighted sample sizes by wave. [file ADD-121-825-s004.docx]

**Contents**

Table 1. Unweighted sample sizes within each survey wave

**Table 1.** Unweighted sample sizes within each survey wave

|  | **Unweighted *n*** | | | | | |
| --- | --- | --- | --- | --- | --- | --- |
| **Wave** | **Total number of participants** | **Risky drinkers** | **Risky drinkers who made ≥1 alcohol reduction attempt** | **Risky drinkers who made ≥1 cost-motivated alcohol reduction attempt** | **Risky drinkers who made ≥1 alcohol reduction attempt solely motivated by cost** |  |
|  |  |  |  |  |  |  |
| Jan-21 | 2403 | 811 | 331 | 33 | 7 |  |
| Feb-21 | 2363 | 655 | 238 | 32 | 2 |  |
| Mar-21 | 2341 | 682 | 230 | 31 | 6 |  |
| Apr-21 | 2394 | 702 | 268 | 35 | 7 |  |
| May-21 | 2239 | 742 | 291 | 33 | 3 |  |
| Jun-21 | 2376 | 763 | 282 | 38 | 9 |  |
| Jul-21 | 2440 | 824 | 279 | 39 | 4 |  |
| Aug-21 | 2192 | 688 | 238 | 32 | 5 |  |
| Sep-21 | 2197 | 751 | 273 | 50 | 9 |  |
| Oct-21 | 2161 | 715 | 234 | 27 | 4 |  |
| Nov-21 | 2480 | 790 | 284 | 39 | 8 |  |
| Dec-21 | 2178 | 682 | 220 | 25 | 3 |  |
|  |  |  |  |  |  |  |
| Jan-22 | 2298 | 740 | 260 | 25 | 4 |  |
| Feb-22 | 2200 | 686 | 233 | 32 | 5 |  |
| Mar-22 | 2607 | 775 | 261 | 34 | 7 |  |
| Apr-22 | 2190 | 713 | 262 | 30 | 3 |  |
| Jun-22 | 2412 | 757 | 238 | 43 | 11 |  |
| Aug-22 | 2369 | 750 | 261 | 41 | 4 |  |
| Oct-22 | 2398 | 799 | 405 | 61 | 15 |  |
|  |  |  |  |  |  |  |
| Jan-23 | 2411 | 699 | 400 | 58 | 11 |  |
| Feb-23 | 2219 | 646 | 402 | 54 | 15 |  |
| Mar-23 | 2397 | 759 | 278 | 51 | 8 |  |
| Apr-23 | 2242 | 657 | 376 | 55 | 11 |  |
| May-23 | 2433 | 738 | 261 | 47 | 5 |  |
| Jun-23 | 2530 | 812 | 265 | 42 | 11 |  |
| Aug-23 | 2411 | 764 | 422 | 61 | 6 |  |
| Oct-23 | 2415 | 761 | 355 | 72 | 15 |  |
| Dec-23 | 2434 | 734 | 250 | 42 | 5 |  |
|  |  |  |  |  |  |  |
| Jan-24 | 2425 | 696 | 280 | 48 | 7 |  |
| Feb-24 | 2375 | 680 | 352 | 52 | 8 |  |
| Mar-24 | 2357 | 713 | 278 | 50 | 6 |  |
| Apr-24 | 2523 | 731 | 374 | 69 | 14 |  |
| Jun-24 | 2425 | 710 | 222 | 38 | 7 |  |
| Aug-24 | 2420 | 700 | 349 | 50 | 7 |  |
| Oct-24 | 2415 | 719 | 352 | 56 | 15 |  |
| Dec-24 | 2431 | 668 | 359 | 51 | 17 |  |
|  |  |  |  |  |  |  |
