# Supplementary material for: Examining changes in the prevalence of cost‐motivated alcohol reduction attempts in the context of a cost‐of‐living crisis and alcohol duty reforms: A population survey of risky drinkers in Great Britain, 2021–2024
Source: Addiction. 2025 Nov 19;121(4):825–38. doi: 10.1111/add.70248 (PMC12980291; doi:10.1111/add.70248)
Supplement: Supplementary file 6 — Appendix S6. Unweighted analyses. [file ADD-121-825-s005.docx]

## Contents

Figure 1: Overall trends

Table 1: Modelled estimates, risky drinkers who made ≥1 past-year alcohol reduction attempt

Figure 2: Trends, subgroups of risky drinkers who made ≥1 past-year alcohol reduction attempt

##
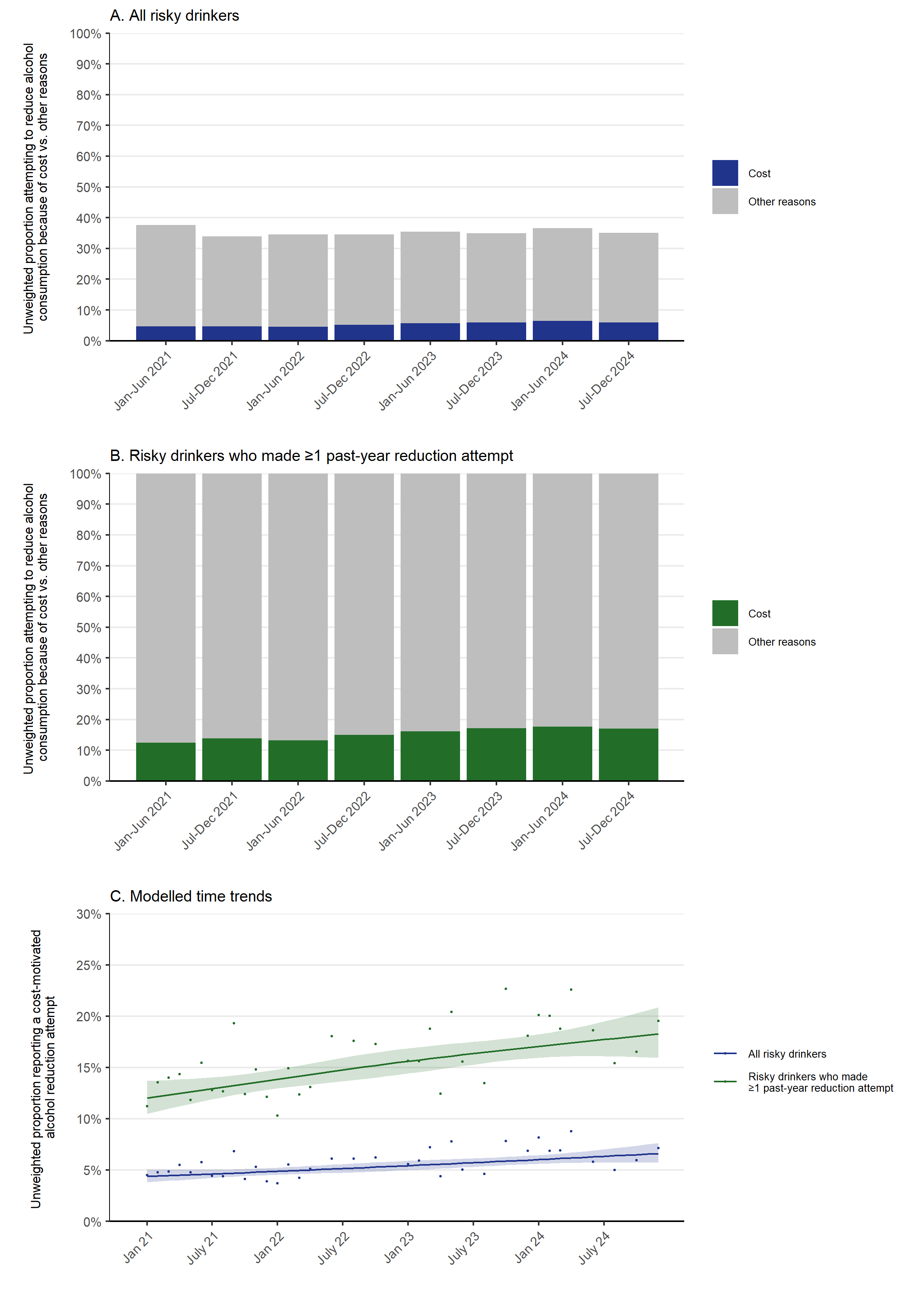
 **Figure 1. Unweighted prevalence of cost-motivated alcohol reduction attempts among risky drinkers (≥18y) in Great Britain, January 2021 to December 2024.**

Panels A and B show unweighted data aggregated across six-month periods, among all risky drinkers and those who attempted to reduce their consumption in the past year, respectively. Bars represent the proportions trying to reduce their alcohol consumption because of cost vs. other reasons.

Panel C shows modelled time trends. Lines represent the modelled unweighted proportion reporting cost-motivated alcohol reduction attempts by monthly survey wave, modelled non-linearly using restricted cubic splines (three knots). Shaded bands represent 95% confidence intervals. Points represent the unmodelled unweighted proportion by month.

## Table 1. Unweighted modelled estimates of changes in the prevalence of cost-motivated alcohol reduction attempts from January 2021 to December 2024 among risky drinkers who made ≥1 past-year alcohol reduction attempt

|  | **Prevalence, % [95%CI]^1^** | | **Prevalence ratio [95%CI]^2^** |
| --- | --- | --- | --- |
|  | **Jan 2021** | **Dec 2024** |  |
|  |  |  |  |
| Overall | 12.0 [10.5–13.7] | 18.3 [15.9–20.9] | 1.52 [1.37-1.68] |
|  |  |  |  |
| Level of risky drinking (AUDIT-C score)^3^ |  |  |  |
| 5 (lowest) | 10.9 [8.2–14.4] | 17.3 [12.9–22.7] | 1.58 [1.04-2.12] |
| 8 | 11.9 [9.8–14.4] | 18.5 [15.2–22.4] | 1.55 [1.17-1.94] |
| 12 (highest) | 17.4 [11.1–26.2] | 21.6 [13.2–33.2] | 1.24 [0.49-1.99] |
|  |  |  |  |
| Nation |  |  |  |
| England | 12.2 [10.6–14.0] | 18.7 [16.2–21.4] | 1.53 [1.27-1.78] |
| Wales/Scotland | 10.3 [6.5–16.0] | 15.2 [9.6–23.2] | 1.47 [0.62-2.32] |
|  |  |  |  |
| Year of age^4^ |  |  |  |
| 18 | 29.9 [22.6–38.4] | 44.2 [34.4–54.5] | 1.48 [1.00-1.95] |
| 25 | 22.2 [18.1–26.9] | 35.2 [29.0–41.8] | 1.58 [1.20-1.97] |
| 35 | 14.3 [12.1–16.8] | 24.4 [20.7–28.6] | 1.71 [1.37-2.05] |
| 45 | 10.0 [8.1–12.3] | 17.3 [13.9–21.3] | 1.73 [1.29-2.18] |
| 55 | 8.4 [6.8–10.2] | 13.4 [10.8–16.5] | 1.60 [1.18-2.02] |
| 65 | 8.5 [6.7–10.7] | 11.5 [9.1–14.5] | 1.36 [0.97-1.76] |
|  |  |  |  |
| Gender |  |  |  |
| Men | 11.1 [9.3–13.3] | 17.5 [14.7–20.8] | 1.57 [1.24-1.91] |
| Women | 13.1 [10.7–16.0] | 19.2 [15.4–23.8] | 1.49 [1.08-1.85] |
|  |  |  |  |
| Social grade |  |  |  |
| ABC1 (most advantaged) | 10.6 [9.0–12.5] | 17.7 [15.0–20.7] | 1.66 [1.33-1.99] |
| C2DE (least advantaged) | 16.0 [12.8–20.0] | 20.9 [16.1–26.7] | 1.30 [0.93-1.68] |
|  |  |  |  |
| Working status |  |  |  |
| Full-time employment/self-employed | 10.3 [8.6–12.4] | 19.1 [16.0–22.7] | 1.85 [1.44-2.26] |
| Part-time employment/unemployed and seeking work/other | 14.5 [12.0–17.5] | 17.0 [13.6–20.9] | 1.17 [0.87-1.47] |
|  |  |  |  |
| Children in the household |  |  |  |
| 0 | 11.8 [10.0–13.7] | 17.0 [14.4–19.9] | 1.44 [1.16-1.73] |
| ≥1 | 12.8 [9.9–16.4] | 22.6 [17.4–28.7] | 1.76 [1.20-2.32] |
|  |  |  |  |
| Smoking status |  |  |  |
| Never | 11.7 [9.7–14.1] | 17.2 [14.0–20.9] | 1.47 [1.12-1.82] |
| Former | 10.3 [8.0–13.2] | 18.6 [14.7–23.3] | 1.80 [1.27-2.33] |
| Current | 17.7 [13.3–23.3] | 21.1 [15.4–28.2] | 1.19 [0.77-1.61] |
|  |  |  |  |
| Past-month psychological distress^5^ |  |  |  |
| No/low | 8.9 [7.1–11.0] | 12.4 [10.9–14.1] | 1.40 [1.03-1.78] |
| Moderate/severe | 18.7 [15.1–22.9] | 21.9 [19.3–24.6] | 1.17 [0.85-1.48] |
|  |  |  |  |

*Table footnotes on next page.*

^1^ Data are unweighted estimates of prevalence in the first and last months in the study period from logistic regression with survey month modelled non-linearly using restricted cubic splines (three knots).

^2^ Prevalence ratio calculated as prevalence in December 2024 (or June 2023, for estimates by history of mental health conditions) divided by prevalence in January 2021 with 95% CIs calculated using bootstrapping (1,000 replications).

^3^ AUDIT-C scores for risky drinkers range from 5 to 12. Modelled estimates are shown for selected scores to illustrate differences. Note that the model used to derive these estimates included data from participants with any score on this scale, not only those with a score of exactly 5, 8, or 12.

^4^ Modelled estimates are shown for selected ages to illustrate differences. Note that the model used to derive these estimates included data from participants of all ages, not only those who were aged exactly 18, 25, 35, 45, 55, or 65 years.

^5^ Data on psychological distress were not collected after June 2023; estimates shown are therefore for January 2021 and June 2023, rather than January 2021 and December 2024.


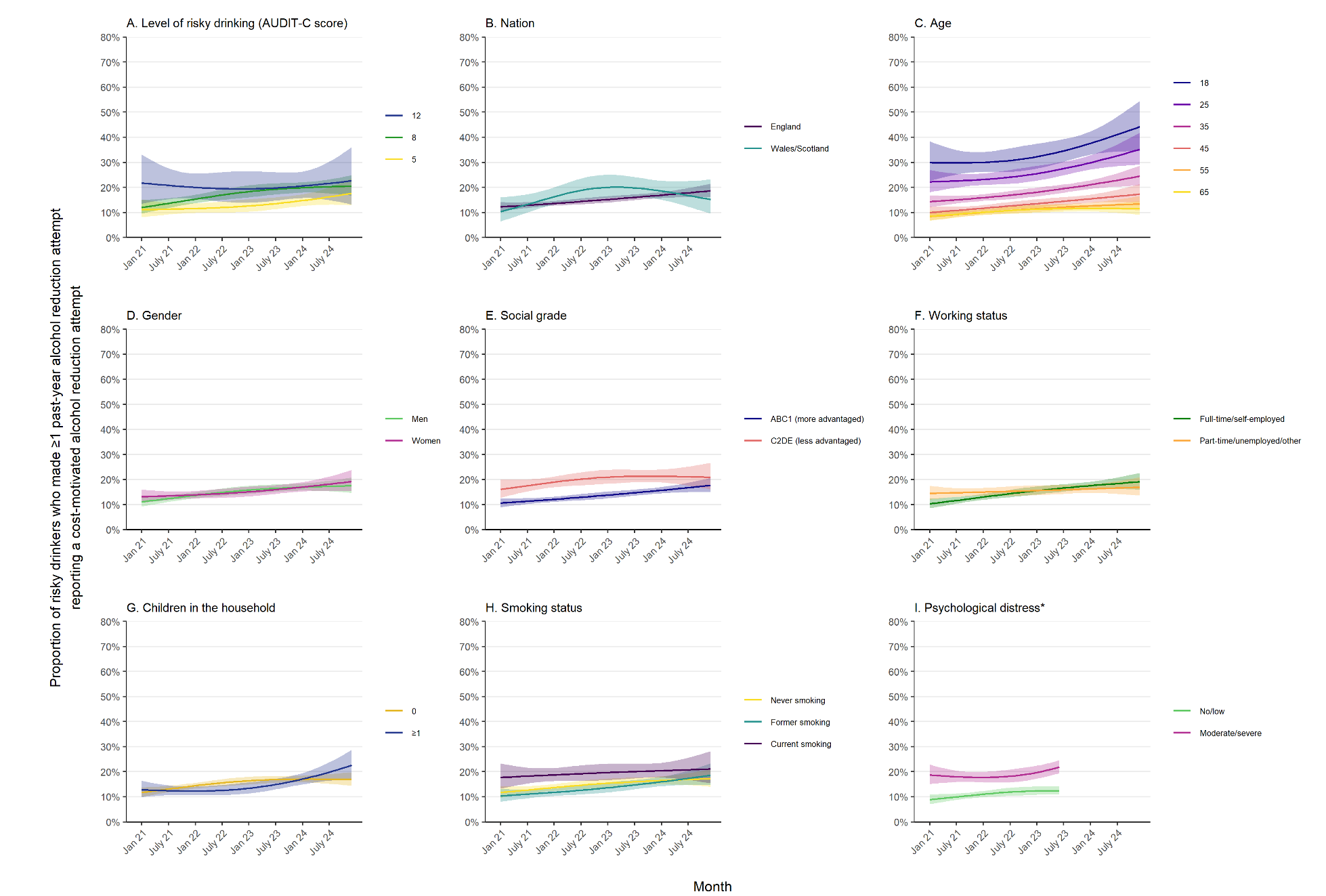


*Figure legend on next page.*

## **Figure 2. Trends in the unweighted prevalence of cost-motivated alcohol reduction attempts among subgroups of risky drinkers (≥18y) in Great Britain who made ≥1 past-year alcohol reduction attempt, January 2021 to December 2024.** Lines represent the modelled unweighted proportion reporting cost-motivated alcohol reduction attempts by monthly survey wave (modelled non-linearly using restricted cubic splines with three knots) and (A) level of risky drinking, (B) nation, (C) age, (D) gender, (E) social grade, (F) working status, (G) children in the household, (H) smoking status, and (I) psychological distress. Shaded bands represent 95% confidence intervals. Points represent the unmodelled unweighted proportion by month. *Data on psychological distress were only available up to June 2023.
